# Diagnostic Performance of Artificial Intelligence in Chest Radiographs Referred from the Emergency Department

**DOI:** 10.3390/diagnostics14222592

**Published:** 2024-11-18

**Authors:** Julia López Alcolea, Ana Fernández Alfonso, Raquel Cano Alonso, Ana Álvarez Vázquez, Alejandro Díaz Moreno, David García Castellanos, Lucía Sanabria Greciano, Chawar Hayoun, Manuel Recio Rodríguez, Cristina Andreu Vázquez, Israel John Thuissard Vasallo, Vicente Martínez de Vega

**Affiliations:** 1 Hospital Universitario QuironSalud Madrid, 28223 Madrid, Spain; anuskafer83@hotmail.com (A.F.A.); rcanoalonso@gmail.com (R.C.A.); anaalvarezvazquez@gmail.com (A.Á.V.); adiaz_14@hotmail.com (A.D.M.); dvdgarcia1995@gmail.com (D.G.C.); lucia.sanabria@quironsalud.es (L.S.G.); chawarhayoun@gmail.com (C.H.); mrmrecio@gmail.com (M.R.R.); vmartinezdevega@malvaluz.com (V.M.d.V.); 2 Faculty of Biomedical and Health Science, Universidad Europea de Madrid, 28670 Madrid, Spain; cristina.andreu@universidadeuropea.es (C.A.V.); israeljohn.thuissard@universidadeuropea.es (I.J.T.V.)

**Keywords:** artificial intelligence, radiography, X-ray, chest, emergency, digital radiography, automatic, detection, diagnosis

## Abstract

Background: The increasing integration of AI in chest X-ray evaluation holds promise for enhancing diagnostic accuracy and optimizing clinical workflows. However, understanding its performance in real-world clinical settings is essential. Objectives: In this study, we evaluated the sensitivity (Se) and specificity (Sp) of an AI-based software (Arterys MICA v29.4.0) alongside a radiology resident in interpreting chest X-rays referred from the emergency department (ED), using a senior radiologist’s assessment as the gold standard (GS). We assessed the concordance between the AI system and the resident, noted the frequency of doubtful cases for each category, identified how many were considered positive by the GS, and assessed variables that AI was not trained to detect. Methods: We conducted a retrospective observational study analyzing chest X-rays from a sample of 784 patients referred from the ED at our hospital. The AI system was trained to detect five categorical variables—pulmonary nodule, pulmonary opacity, pleural effusion, pneumothorax, and fracture—and assign each a confidence label (“positive”, “doubtful”, or “negative”). Results: Sensitivity in detecting fractures and pneumothorax was high (100%) for both AI and the resident, moderate for pulmonary opacity (AI = 76%, resident = 71%), and acceptable for pleural effusion (AI = 60%, resident = 67%), with negative predictive values (NPV) above 95% and areas under the curve (AUC) exceeding 0.8. The resident showed moderate sensitivity (75%) for pulmonary nodules, while AI’s sensitivity was low (33%). AI assigned a “doubtful” label to some diagnoses, most of which were deemed negative by the GS; the resident expressed doubt less frequently. The Kappa coefficient between the resident and AI was fair (0.3) across most categories, except for pleural effusion, where concordance was moderate (0.5). Our study highlighted additional findings not detected by AI, including 16% prevalence of mediastinal abnormalities, 20% surgical materials, and 20% other pulmonary findings. Conclusions: Although AI demonstrated utility in identifying most primary findings—except for pulmonary nodules—its high NPV suggests it may be valuable for screening. Further training of the AI software and broadening its scope to identify additional findings could enhance its detection capabilities and increase its applicability in clinical practice.

## 1. Introduction

Over the past few years, the application of artificial intelligence (AI), particularly deep learning (DL), in medical imaging examinations has increased significantly.

AI-based software is revolutionizing radiology, with applications that continue to expand. Among the most notable extrathoracic applications are the detection of suspicious lesions in mammograms, multiple sclerosis and Alzheimer’s disease in brain MRIs, hemorrhages in cranial CT scans, stroke in CT angiography, fractures in bone X-rays, and injuries to ligaments, menisci, and cartilage in knee MRIs [1,2,3,4,5,6,7,8,9,10,11,12]. Among all subfields of medical imaging, thoracic imaging is likely the subfield where AI applications have been most extensively studied [13]. In the context of thoracic pathology, CAD systems are capable of detecting a wide range of conditions, including pulmonary nodules, pneumonia (including COVID-19), tuberculosis, interstitial lung disease, pulmonary edema, pneumothorax, pleural effusion, mediastinal masses, and acute aortic syndrome, among others, in both chest radiographs and CT scans [14,15]. Additionally, AI is employed to enhance image quality and speed in both MRI and CT scans [16,17,18].

With the increasing number of AI applications in radiology, it will be essential to provide adequate training for radiologists on when and how to incorporate AI suggestions into their readings. Moreover, radiologists must actively participate in the evaluation of AI tools, as these technologies can support their daily work and enable them to focus on more complex tasks.

Assisting clinicians—including both radiologists and emergency physicians—in the evaluation of chest X-rays, AI software has the potential to improve diagnostic accuracy and streamline workflows. This facilitates prioritizing reports for patients with significant findings and reduces the time required for interpretation [13,19]. Nevertheless, the effective integration of AI tools with human decision-making processes remains an area of ongoing investigation [20].

AI-based devices must demonstrate higher accuracy than their intended users to establish a justified role in diagnostic imaging. This is crucial, since less experienced human observers are more prone to availability bias, heightening the risk of being influenced by inaccurate AI-generated advice [15].

The main objectives of this study are as follows:

▪ To evaluate the sensitivity and specificity of AI software and a radiology resident in the evaluation of chest X-ray examinations referred from the ED, in comparison with a senior radiologist, regarded as the gold standard (GS).
▪ To assess the concordance rate between AI and the resident.

The secondary objectives are as follows:

▪ To describe the frequency of doubtful cases in each category and how many of them are considered positive by the GS.
▪ To evaluate other variables that AI is not trained to detect, in order to analyze its weaknesses and potential areas for improvement in diagnosis.

## 2. Materials and Methods

We conducted a double-blind, observational, descriptive, cross-sectional, retrospective, and single-center study, evaluating thoracic radiographs obtained with digital systems from patients referred from the ED at Hospital Universitario QuironSalud Madrid between October 15th and November 15th, 2022. Data were retrieved from the hospital’s radiology database and analyzed retrospectively.

**Figure 1:**
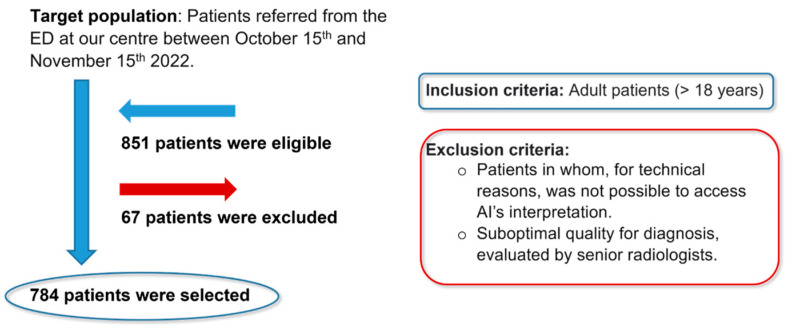
Flowchart of the study population.

Inclusion criteria were radiographs from adult patients (>18 years) regardless of gender, while exclusion criteria included radiographs in which, due to technical reasons, AI interpretation was not accessible, and radiographs of suboptimal diagnostic quality, as assessed by senior radiologists. Of the 851 eligible patients, 67 were excluded, resulting in a final sample of 784 patients (Figure 1).

**Figure 2:**
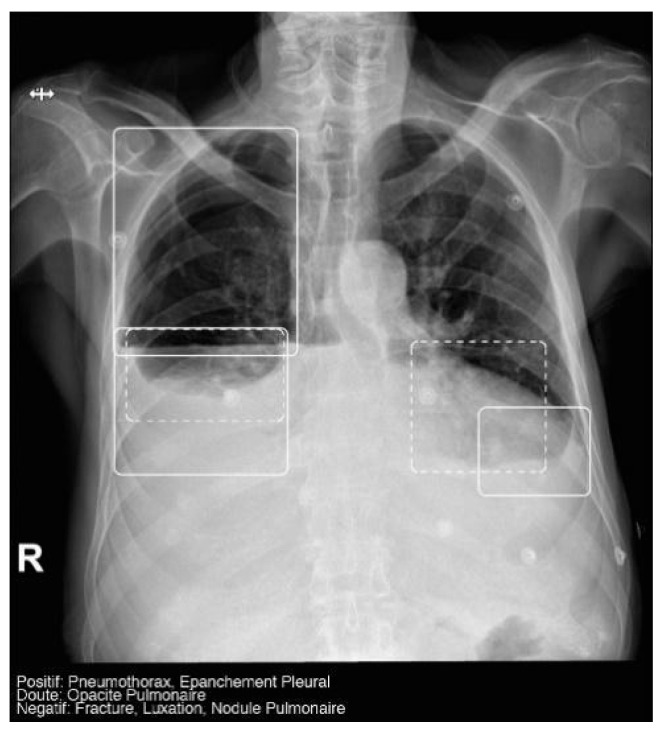
Chest radiograph with AI analysis, which detected a positive right pneumothorax, positive bilateral pleural effusion (both outlined with continuous boxes), and doubtful bibasilar opacities (outlined with discontinuous boxes).

The AI software (Arterys Chest MICA v29.4.0, developed by Arterys, a company based in Paris, France) was the World’s First Online Medical Imaging Platform 100% Cloud native, powered by AI and FDA cleared. It was a clinical application (CE marked as a Class IIa medical device) designed to process thoracic radiographic series and identify five imaging findings (categorical variables): pulmonary nodule, pulmonary opacity, pleural effusion, pneumothorax, and fracture. Each detected finding was localized in the image using a bounding box and was assigned a confidence label, either “positive” (continuous line) or “doubtful” (dashed line). Moreover, the algorithm provided a list of findings not detected in the current radiographic view (Figure 2). All findings were detected by a deep learning model that processed all radiographic views included in the series.

The AI results were integrated into the institutional picture archiving and communication system (PACS) and the clinicians’ image viewer, being displayed in a secondary capture apart from the original radiograph. In practice, radiologists first evaluated the chest X-ray images and then reviewed the AI results on the reading workstation to assist in completing the radiology report. When the requesting physicians opened the chest X-ray in the viewer, they could also see the AI’s analysis.

The deep learning algorithm within the AI Chest product consisted of a convolutional neural network that detected the aforementioned findings in each radiographic view. The model was trained and validated using 1,262,467 and 157,181 radiographic images, respectively, and was further calibrated using an independent dataset of 4759 images. The data used for model development were sourced from a multicentric database, which included both pediatric and adult patients.

**Figure 3:**
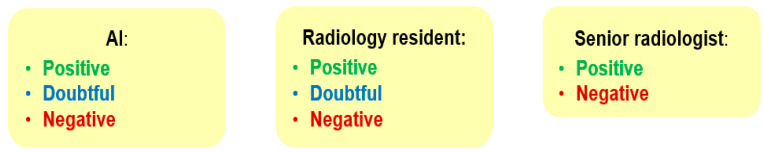
Possible results of chest X-ray assessment.

All images were reviewed using a PACS system by three independent readers: two radiology residents and a senior radiologist, who entered the data into a spreadsheet program. One radiology resident (with 1 year of training) independently recorded the AI’s readings. The second radiology resident (with 2 years of training) qualitatively evaluated each examination, assessing the presence of the aforementioned findings and providing a positive, negative, or doubtful result. The senior radiologist (with over 10 years of experience) also independently reviewed each examination, offering either a positive or negative result. Figure 3 shows the possible X-ray assessment results according to the different readers. None of the readers specified the locations of findings within the different lung zones, and all were blinded to both clinical information and the AI’s readings. Finally, the data were fully de-identified.

We described the following:

- The diagnostic performance of AI and the resident against the GS: evaluating sensitivity (Se), specificity (Sp), predictive values, area under the curve (AUC), and 95% confidence intervals (CI).
- The frequency of doubtful results: determining the occurrence of doubtful findings and their classification by the GS.
- The frequency of other variables that AI did not detect but could have an impact on diagnosis: mediastinal abnormalities, surgical material, and other pulmonary findings.

Regarding statistical analysis, we stated the following:

- Qualitative variables were described in absolute (*n*) and relative (%) frequencies.
- Continuous variables as means (±standard deviation) or medians (interquartile range).
- For AI validation, contingency tables were used to calculate sensitivity, specificity, predictive values, area under the curve (AUC) (compared with GS), and 95% confidence intervals (CI).
- The Kappa coefficient was used to evaluate interobserver agreement between the resident and AI.
- All statistical analyses were calculated using SPSS statistical software, Version 25.0 (IBM).

Our study was approved by the Ethics Committee of our institutional review board (Hospital Fundación Jiménez Díaz; Grupo QuironSalud) on 10/01/2023 (no 01/23) and again on 28/02/2023 (no 04/23) with the code EO017-23_HUQM.

## 3. Results

### 3.1. Statistical Results

#### 3.1.1. Demographics

The final dataset comprised 784 patients, with a slightly higher proportion of women than men (51.79% vs. 48.21%), and a median age of 58 years. Most radiographs (86.10%) included two projections (posteroanterior and lateral) and 90.18% were of optimal quality (Table 1).

#### 3.1.2. Prevalence

Prevalence was based on the GS diagnosis and was adjusted by the confidence interval, making it more precise. The most prevalent finding was pulmonary opacity (14.29%), while pneumothorax was the least prevalent (0.38%). Interestingly, the prevalence of variables not analyzed by AI was higher (16.33–20.82%) compared with those that were analyzed (Table 2).

#### 3.1.3. Diagnostic Performance

In terms of diagnostic efficacy, we emphasized the statistical parameters of sensitivity, negative predictive value, and area under the curve (AUC), as these are the most critical for measuring the accuracy of a screening method, such as chest radiography in the emergency department. The AUC is an effective and combined measure of sensitivity and specificity for assessing the inherent validity of a diagnostic test [21]. An AUC greater than 0.8 is statistically considered good for screening purposes [22,23,24]. As for the AI’s and the resident’s diagnostic performance, cases with doubtful diagnoses were excluded to calculate sensitivity, specificity, PPV, NPV, and AUC.

AI exhibited high sensitivity (100%) for fractures and pneumothorax, moderate for pulmonary opacities (76%), reasonable for pleural effusions (60%), and low for pulmonary nodules (33%) (Table 3). When AI doubted, only a few of these doubtful diagnoses were considered positive by the GS, with the exception of pulmonary nodules.

##### Fracture

Prevalence was low at 1.28%. AI achieved excellent results, with a sensitivity and NPV of 100% and an AUC of 0.99. Only 15.79% of the doubtful results were really positive.

##### Pneumothorax

Prevalence was very low at 0.38%. AI also achieved perfect sensitivity and NPV (100%). Only 11.11% of the doubtful diagnoses were really positive.

##### Pulmonary Nodule

Prevalence was also low at 2.55%. AI’s sensitivity was relatively low at 33.3%, but NPV was high (98.9%). Notably, 50% of doubtful pulmonary nodules were actually positive.

##### Pulmonary Opacity

Its confidence intervals were the best, since its prevalence was the highest at 14.29%. AI doubted often and only 17% of these doubtful findings were really positive. AI had moderate sensitivity (75.6%), high NPV (96.2%), and AUC (0.86).

##### Pleural Effusion

Prevalence was moderate at 9.1%, and 42% of doubtful results were really positive. Sensitivity was relatively poor (59.7%), though NPV was high (96.5%) and AUC was 0.79.

**Figure 4:**
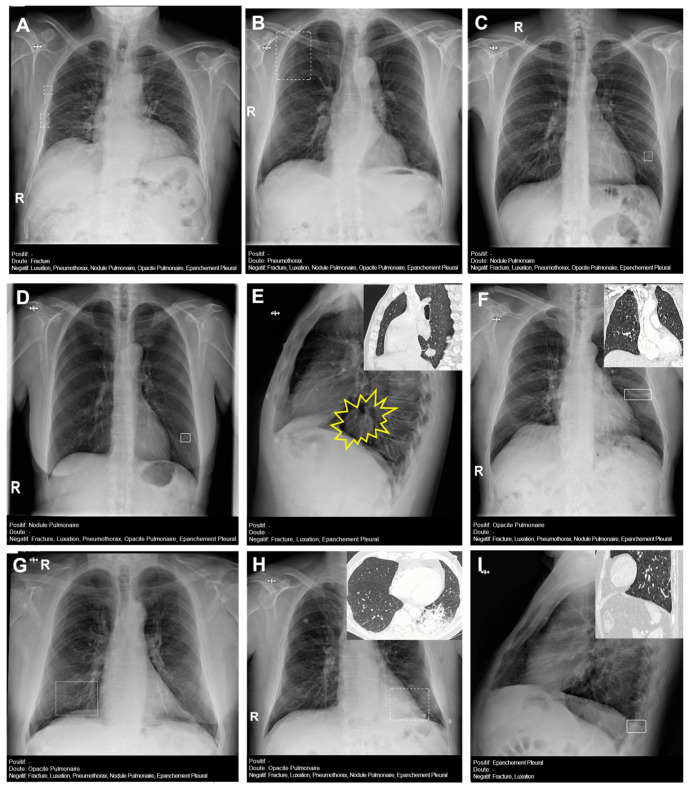
Chest radiographs with AI analysis (and **E**,**F**,**H**,**I** with the associated sagittal (**E**,**I**), coronal (**F**), and axial (**H**) CT images). (**A**) False doubtful right fractures outlined with discontinuous boxes (they are chronic). (**B**) False doubtful right pneumothorax outlined with a discontinuous box. (**C**) False doubtful left lower lobe nodule outlined with a discontinuous box. (**D**) False positive left lower lobe nodule outlined with a continuous box. (**E**) False negative left lower lobe nodule; there is a nodule (outlined in yellow). (**F**) False positive left pulmonary opacity outlined with a continuous box (it is a laminar atelectasis). (**G**) False doubtful right lower lobe opacity outlined with a discontinuous box (it is normal pulmonary vascularization). (**H**) True doubtful left lower lobe opacity outlined with a discontinuous box (retrocardiac infection). (**I**) False positive pleural effusion outlined with a continuous box (it is a subtle non-pathological erasure of the posterior costophrenic angle).

Figure 4 presents several examples of chest radiographs analyzed by the AI.

#### 3.1.4. Resident

The resident doubted less in all categories and had the same sensitivity for fractures and pneumothorax (100%), much better for pulmonary nodule (75%), slightly better for pleural effusion (67%), and slightly lower for pulmonary opacity (71%).

#### 3.1.5. Other Variables

**Figure 5:**
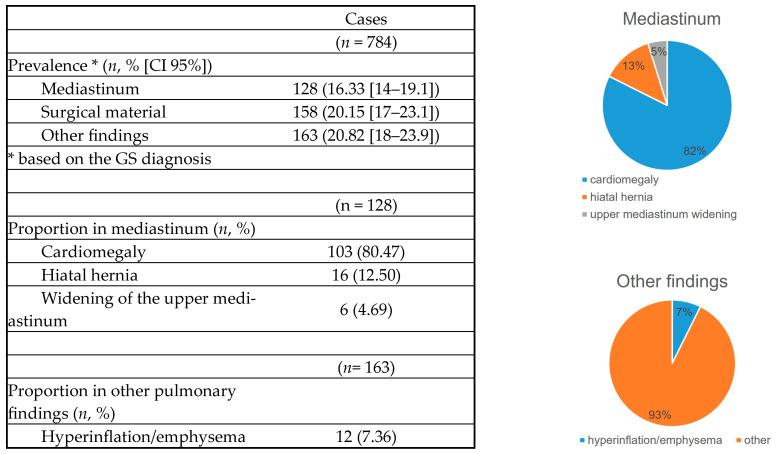
Extra items.

**Figure 6:**
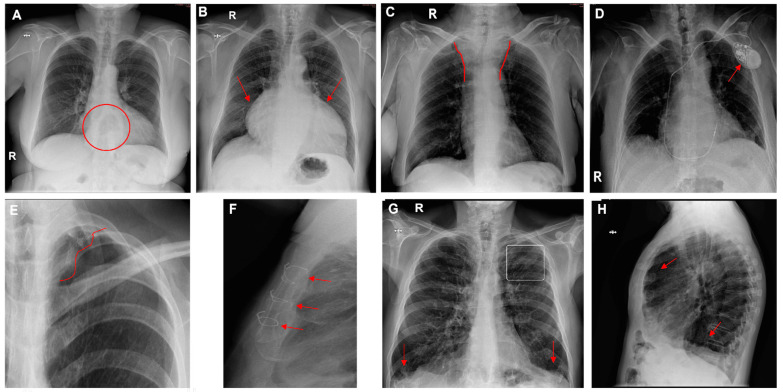
Chest radiographs with additional findings highlighted by red circles, arrows, or lines. (**A**) Hiatal hernia, (**B**) cardiomegaly, (**C**) widening of the superior mediastinum, (**D**) heart pacemaker, (**E**) pulmonary surgical material, (**F**) sternal surgical material, (**G**,**H**) pulmonary hyperinflation (**G**—flattening of the diaphragms and enlarged retrosternal space; **H**—enlarged retrocardiac spaces).

The prevalence of other variables included 16% for mediastinal abnormalities (cardiomegaly, hiatal hernia, widening of the superior mediastinum), 20% for surgical material (staples, metal valves, stents), and 20% for other pulmonary findings (pulmonary hyperinflation). Cardiomegaly was the most frequent finding (80%). Hyperinflation was found in only 7% (Figure 5 and Figure 6).

#### 3.1.6. Concordance Rate for AI and the Resident

**Figure 7:**
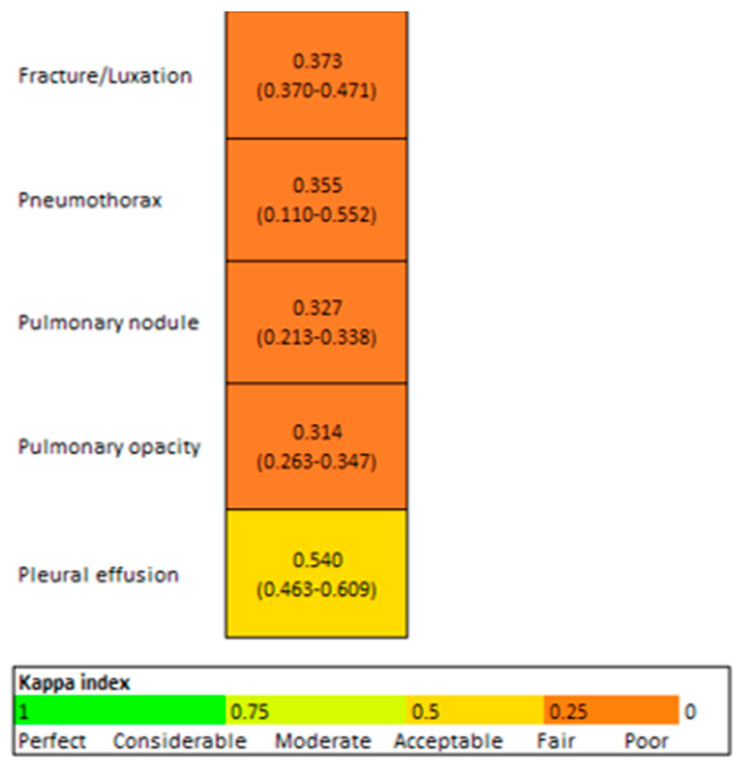
Concordance AI/Resident (95% CI).

The Kappa coefficient between the resident and AI was fair (0.3) for all variables, except for pleural effusion, which was moderate (0.5) (Figure 7).

## 4. Discussion

Chest X-ray constitutes the most commonly performed radiologic examination worldwide, particularly in the emergency department (ED), due to its availability, low cost, low radiation, and portability [14,15,25]. It is essential for the screening, diagnosis, and management of a wide range of conditions. Although chest X-ray reporting is considered a basic radiological skill, it is an inherently difficult and subjective task and it requires a more gradual learning curve because of its limited spatial resolution and the noise caused by overlapping anatomical structures, unlike computed tomography (CT) scans [25,26,27]. Moreover, this challenge is heightened by the fact that less time is currently devoted to teaching it during radiology residency training due to the advancements in other imaging techniques. It has been reported that the chest radiograph is the most frequently misinterpreted radiograph, especially in the ED [28].

The integration of AI-based computer-aided detection (CAD) systems is particularly beneficial in environments where the following difficulties are present [13,19]:

- Remote work by radiologists: in contexts where radiologists work from locations separate from imaging facilities, AI can provide crucial diagnostic support for remote interpretation.
- Shortage of radiologists: in regions or during peak hours where there is a shortage of radiologists, unmet reporting demands can be mitigated by integrating AI as a complementary diagnostic tool [29,30].
- High demand for radiological examinations: with the increasing volume of radiological examinations, AI can help manage and streamline the workload by providing rapid preliminary assessments. This not only helps in reducing significantly the burden on radiologists but also speeds up the diagnostic process.
- Direct evaluation by emergency physicians: in many EDs, X-rays are evaluated directly by treating emergency physicians (who rely on personal experience and basic skills) rather than by radiologists. Various studies have reported discrepancies between radiograph interpretations in the emergency department and those made by radiologists [28]. AI has the potential to enhance diagnostic accuracy and efficiency in these contexts, assisting emergency physicians in making informed clinical decisions.
- AI can serve as a valuable resource for radiologists in training, particularly when senior radiologists are not available, offering diagnostic support and enhancing learning opportunities.

Advancements in computer vision and AI hold great potential for healthcare, especially in diagnostic specialties like radiology [30]. With the rise in deep learning and AI applications in medical imaging, there is growing interest in developing AI algorithms for chest radiographs to assist clinicians in accurately and efficiently detecting key radiographic findings. Many previous studies have used curated datasets that are not reflective of real clinical practice, as they contain artificially high rates of pathological findings compared with the real world. This may result in inflated accuracy metrics [31]. In contrast, our study analyzed a consecutive series of chest X-rays, better representing routine clinical practice. Our findings suggest that AI is effective in detecting fractures, pneumothorax, pulmonary opacities, and pleural effusions, although it has limitations in accurately identifying pulmonary nodules.

In our study, AI achieved a sensitivity of 100% (95% CI, 59–100%), specificity of 98.7% (95% CI, 97.6–99.4%), and AUC of 0.993 (95% CI, 0.989–0.997) for fractures. Lee et al. reported lower sensitivity (87%), specificity (83%), and a slightly lower AUC (89%) [32]. Similarly, Sun et al. found lower sensitivity (82.2%) [33].

For pneumothorax, our study demonstrated AI sensitivity of 100% (95% CI, 15.8–100%), specificity of 100% (95% CI, 99.5–100%), and an AUC of 1 (95% CI, 1–1). In comparison, Bennani et al. reported a slightly lower sensitivity of 88.6%, while maintaining a nearly identical specificity of 99.0% [26]. Ahn et al. obtained results similar to ours, with a sensitivity of 98.8%, specificity of 98.6%, and an AUROC of 0.999 [34]. Van Beek, however, reported lower sensitivity (83.3%) but comparable specificity (97.8%) and a slightly lower AUC (95.4%) [31].

For pulmonary opacity, our study reported AI sensitivity of 75.6% (95% CI, 64.9–84.4%), specificity of 95.3% (95% CI, 93.1–96.6%), and AUC of 0.855 (95% CI, 0.807–0.902). Bennani et al. obtained slightly higher sensitivity (81.5%) and similar specificity (92.0%) [26]. Ahn et al. reported higher sensitivity (88.7%) but lower specificity (72.8%) with a similar AUROC (0.880) [34]. Van Beek’s results showed higher sensitivity (88.6%), and lower specificity (79.2%), with a slightly higher AUC (0.90) [31].

For pleural effusion, our study showed AI sensitivity of 59.7% (95% CI, 46.4–71.9%), specificity of 98.9% (95% CI, 97.8–99.5%), and an AUC of 0.793. Bennani et al. reported higher sensitivity (89.0%, the highest in their study) but slightly lower specificity (93.6%) [26]. Ahn et al. also reported higher sensitivity (87.2%) with similar specificity (96%) and a higher AUC (0.983) [34]. Van Beek demonstrated much higher sensitivity (78.4%), similar specificity (94.2%), and a much higher AUC (0.95) [31].

The low sensitivity for pulmonary nodules (33.3%; 95% CI, 9.92–65.1%) in our study aligns with other research. Bennani et al. and Ahn et al. also found low AI sensitivity for lung nodules (60.3% and 81.6%, respectively) [26,34]. However, studies such as Van Beek et al. (79.4%) and Nam et al. (80.7%), demonstrated better performance with CAD systems for chest X-ray nodule detection [27,31]. Our study showed high specificity (99.6%; 95% CI, 98.8–99.9%) and an AUC of 0.665 (95% CI, 0.525–0.804). Ahn et al. reported lower specificity (0.731) but a higher AUROC (0.858), while Nam et al. had similar specificity (95.2%) and AUC (0.92–0.99) for malignant nodules. Van Beek reported lower specificity (84.8%) and a higher AUC (0.881). It is important to emphasize that most pulmonary nodules are benign and do not represent urgent findings. Chest X-ray is significantly less sensitive than CT for detecting pulmonary nodules and is not typically used as a screening tool for them [13]. In clinical practice, pneumologists recommend low-dose chest CT for patients with cancer risk factors.

Low prevalence, such as with pneumothorax and pulmonary nodules, can affect diagnostic performance by widening confidence intervals and thus reducing precision. Regarding limitations, AI cannot differentiate between acute and chronic fractures (e.g., callus formation), leading to potential false positives. Additionally, most doubtful pulmonary opacities identified by AI correspond to normal vascularization in the right lower or middle lobe. While AI accurately detects large and moderate pleural effusions, it struggles with subtle effusions, often mistaking them for non-pathological blurring of the costophrenic angle. These issues highlight a key challenge in accepting computer-aided diagnosis in chest radiography (the large number of false positives requiring further assessment) [27].

In our study, the resident showed the same sensitivity as AI for fractures and pneumothorax (100% [95% CI, 69.2–100%] and 100% [95% CI, 29–100%], respectively), significantly higher sensitivity for pulmonary nodules (75% [95% CI, 50.9–91.3%]), slightly improved sensitivity for pleural effusion (67.1% [95% CI, 54.6–77.9%]), and slightly lower sensitivity for pulmonary opacity (71.2% [95% CI, 61.4–79.6%]). Ahn et al. reported lower resident sensitivity for nodules (60.0%), similar sensitivity for pulmonary opacity (70.1%), higher sensitivity for pleural effusion (85.9%), and lower sensitivity for pneumothorax (73.5%) [34]. Bennani et al. found that AI exhibited higher sensitivity across all abnormality types compared with unassisted readers, with the largest difference seen for pneumothorax (36.2%). AI assistance led to increases in sensitivity across different experience levels, including general radiologists and residents [26]. Wu et al.’s algorithm showed no significant difference in sensitivity between AI (71.6%) and third-year residents (72.0%) [35], while Yoo et al. demonstrated a 10% increase in sensitivity for cancer detection among residents [36].

Our study focused exclusively on comparing the diagnostic accuracy of the resident and AI independently, using a senior radiologist as the GS. In contrast, other studies evaluated radiologists’ performance with and without AI assistance, which complicated direct comparisons. The resident’s lower level of doubt, compared with that of AI, significantly impacted the prevalence of findings in the statistical analysis and influenced concordance rate calculations (Cohen’s Kappa), resulting in fair agreement in our study. In contrast, Kim et al. reported a concordance rate of 86.8% between radiologists and AI in clinical practice [30]. Ahn et al. found that AI-aided interpretation improved reader sensitivity and AUROC for all target findings without negatively affecting specificity [34].

Concordance between the senior radiologist, considered the gold standard, and the AI system was not calculated because the senior radiologist’s assessment served as the reference standard for evaluating AI’s diagnostic performance. Calculating concordance between these two would compromise the objectivity of the evaluation, as the gold standard was not an independent diagnostic outcome but rather the benchmark against which the AI was measured. Thus, any concordance calculation would lack validity, as it would essentially compare the AI’s output to its intended standard rather than independently assessing its accuracy.

Both AI and the resident demonstrated NPVs > 95% and AUCs > 0.8 (with the exception of pulmonary nodules), along with high 95% CIs, indicating statistically robust performance. This suggests that AI could serve as an effective screening tool in the emergency department by efficiently identifying non-pathological chest radiographs, thereby allowing radiologists to prioritize reports with abnormal findings, particularly those of clinical urgency, such as pneumothorax [34]. However, since most findings classified as doubtful by AI were not true positives, emergency physicians should consult radiologists for these cases. They should also remain aware of AI’s tendency to overestimate findings such as fractures, right lower lobe pulmonary opacities, and mild pleural effusions, and approach these findings with caution. They should reject false-positive findings detected by the AI while benefiting from accepting true-positive ones [34].

Further training of the AI software with a larger patient dataset to improve its sensitivity in detecting pulmonary nodules and reduce false positives for fractures, pulmonary opacities in the middle and right lower lobes, and subtle pleural effusions, along with broadening its scope to identify additional findings, could enhance its detection capabilities and increase its applicability in clinical practice.

While other studies evaluated the time savings associated with AI-assisted image interpretation [26,34], our research focused on diagnostic efficacy and examined findings that the software was not trained to detect, such as cardiomegaly, mediastinal widening, hiatal hernia, pulmonary hyperinflation, and post-surgical materials. Ahn et al. evaluated similar non-target findings like cardiac silhouette, bone fractures, pleural thickening, atelectasis, and pericardial calcifications [34], as did Van Beek, who assessed atelectasis, fibrosis, calcification, cardiomegaly, mediastinal widening, and pneumoperitoneum [31]. We evaluated the potential impact of integrating these clinically relevant findings to enhance daily clinical practice. By assessing pulmonary, skeletal, and mediastinal findings, we believe that the role of artificial intelligence could closely mirror that of a radiologist or emergency physician during the initial assessment of a patient. However, further research is needed to ensure that AI provides equivalent results in real-world prospective studies.

### Limitations

- We evaluated only a small sample of adult patients over a brief period.
- We used a senior radiologist as the gold standard, despite the inherent subjectivity [31,34], without incorporating an additional objective confirmatory test, such as chest CT [26,37,38].
- The added value of AI in radiology should be assessed not only through statistical parameters like sensitivity and specificity, but also by evaluating its impact on workflow efficiency, patient management improvements, and enhancing radiologists’ work–life balance.
- Focusing on five specific abnormalities rather than analyzing all possible abnormalities on a chest X-ray, while also disregarding the patient’s clinical information, does not accurately reflect daily clinical practice.
- The low prevalence of pneumothorax and pulmonary nodules resulted in broad confidence intervals (and, consequently, reduced statistical precision), which may affect Kappa values. Other studies, such as Bennani et al., addressed this issue by having a senior thoracic radiologist select radiographs to create a balanced dataset that included both normal and abnormal images of varying complexity [26].
- The exclusion of doubtful cases in the statistical analysis led to worse results. However, it is still useful in clinical practice, since it alerts that the reliability is lower.
- We independently compared the diagnostic accuracy of AI software with that of manual reading. However, several factors seem to limit the ability of AI-based systems to diagnose pathologies in radiology without human involvement. A more accurate reflection of the clinical setting might be achieved through an observer test, where the algorithm is used as a second or concurrent reader alongside radiologists, considering the impact of human–machine interaction and final human decision making.
- Finally, the concordance of AI was evaluated with only one radiologist resident, which may limit the generalizability of the findings.

## 5. Conclusions

AI is a valuable tool for detecting various chest X-ray abnormalities, with high sensitivity for fracture and pneumothorax (100%), moderate for pulmonary opacity (76%), and reasonable for pleural effusion (60%), with the exception of pulmonary nodules (low sensitivity: 33%). Its high NPV suggests that AI could be implemented as a screening tool in the emergency department, efficiently identifying non-pathological chest radiographs and enabling radiologists to prioritize abnormal cases. When AI doubts, most findings are not truly positive. Further training of AI to detect additional abnormalities could enhance its utility. Concordance between AI and the resident is fair (K = 0.3).

## Figures and Tables

**Table 1:** Study sample’s characteristics.

		Cases
		(*n* = 784)
Patient’s age median [Q1; Q3]		58 [44–74]
Patient’s sex (*n*, %)		
	Man	378 (48.21)
	Woman	406 (51.79)
Number of projections (*n*, %)		
	1	109 (13.9)
	2	675 (86.10)
X-ray quality (*n*, %)		
	Optimal	707 (90.18)
	Fair	77 (9.82)

Q1: First quartile = percentile 25. Q3: Third quartile = percentile 75.

**Table 2:** Findings’ prevalence.

	Cases
	(*n* = 784)
Prevalence * (*n*, % [CI 95%])	
Fracture/Luxation	10 (1.28 [0.61–2.33])
Pneumothorax	3 (0.38 [0.079–1.1])
Pulmonary nodule	20 (2.55 [1.6–3.93])
Pulmonary opacity	112 (14.29 [11–16.2])
Pleural effusion	71 (9.06 [7.1–11.3])
Mediastinum	128 (16.33 [14–19.1])
Surgical material	158 (20.15 [17–23.1])
Other findings	163 (20.82 [18–23.9])

* based on the GS diagnosis. * prevalence adjusted by confidence interval is more precise.

**Table 3:** Diagnostic performance.

		Overall Cases	
		(*n* = 784)	
		Resident’s Diagnostic Performance	AI’s Diagnostic Performance
Fracture (doubtful diagnoses/certain ratio)		0/784	19/765
Doubtful diagnoses/% positive ratio		DA	19/15.79
	Sensitivity (%, 95% CI)	100 (69.2–100)	100 (59–100)
	Specificity (%, 95% CI)	99.9 (99.3–100)	98.7 (97.6–99.4)
	PPV (%, 95% CI)	90.9 (58.7–99.8)	41.2 (18.4–67.1)
	NPV (%, 95% CI)	100 (99.5–100)	100 (99.5–100)
	AUC (95% CI)	0.999 (0.998–1)	0.993 (0.989–0.997)
Pneumothorax (doubtful diagnoses/certain ratio)		0/784	9/775
Doubtful diagnoses/% positive ratio		DA	9/11.11
	Sensitivity (%, 95% CI)	100 (29–100)	100 (15.8–100)
	Specificity (%, 95% CI)	100 (99.5–100)	100 (99.5–100)
	PPV (%, 95% CI)	100 (29.2–100)	100 (15.8–100)
	NPV (%, 95% CI)	100 (99.5–100)	100 (99.5–100)
	AUC (95% CI)	1 (1–1)	1 (1–1)
Pulmonary nodule (doubtful diagnoses/certain ratio)		4/780	16/768
Doubtful diagnoses/% positive ratio		4/0	16/50
	Sensitivity (%, 95% CI)	75 (50.9–91.3)	33.3 (9.92–65.1)
	Specificity (%, 95% CI)	99.3 (98.5–99.8)	99.6 (98.8–99.9)
	PPV (%, 95% CI)	75 (50.9–91.3)	57.1 (18.4–90.1)
	NPV (%, 95% CI)	99.3 (98.5–99.8)	98.9 (97.6–99.5)
	AUC (95% CI)	0.872 (0.774–0.969)	0.665 (0.525–0.804)
Pulmonary opacity (doubtful diagnoses/certain ratio)		16/768	170/614
Doubtful diagnoses/% positive ratio		16/50	170/17.65
	Sensitivity (%, 95% CI)	71.2 (61.4–79.6)	75.6 (64.9–84.4)
	Specificity (%, 95% CI)	99.1 (98–99.7)	95.3 (93.1–96.6)
	PPV (%, 95% CI)	92.5 (84.4–97.2)	71.3 (60.6–80.5)
	NPV (%, 95% CI)	95.6 (93.8–97)	96.2 (94.2–97.7)
	AUC (95% CI)	0.851 (0.807–0.895)	0.855 (0.807–0.902)
Pleural effusion (doubtful diagnoses/certain ratio)		12/772	21/763
Doubtful diagnoses/% positive ratio		12/8.33	21/42.86
	Sensitivity (%, 95% CI)	67.1 (54.6–77.9)	59.7 (46.4–71.9)
	Specificity (%, 95% CI)	97.4 (96–98.5)	98.9 (97.8–99.5)
	PPV (%, 95% CI)	72.3 (59.8–82.7)	82.2 (67.9–92)
	NPV (%, 95% CI)	96.7 (95.2–97.9)	96.5 (94.9–97.7)
	AUC (95% CI)	0.823 (0.767–0.879)	0.793 (0.731–0.854)

CI: Confidence interval. DA: Does not apply.

## Data Availability

The original data presented in the study are openly available in FigShare at doi:10.6084/m9.figshare.27138981.
